# Bazedoxifene Suppresses Intracellular Mycobacterium tuberculosis Growth by Enhancing Autophagy

**DOI:** 10.1128/mSphere.00124-20

**Published:** 2020-04-08

**Authors:** Qi Ouyang, Kehong Zhang, Dachuan Lin, Carl G. Feng, Yi Cai, Xinchun Chen

**Affiliations:** a Guangdong Provincial Key Laboratory of Regional Immunity and Diseases, Department of Pathogen Biology, School of Medicine, Shenzhen University, Shenzhen, China; b Department of Pharmaceutical/Medicinal Chemistry, Institute of Pharmacy, Friedrich-Schiller-University Jena, Jena, Germany; c Immunology and Host Defense Group, Department of Infectious Diseases and Immunology, Faculty of Medicine and Health, University of Sydney, Sydney, New South Wales, Australia; Washington University School of Medicine in St. Louis

**Keywords:** *Mycobacterium tuberculosis*, autophagy, bazedoxifene, host-directed therapy

## Abstract

Tuberculosis (TB) is still the leading killer caused by Mycobacterium tuberculosis infection. There is a clear need for new treatment strategy against TB. It has been reported that tamoxifen, known as a selective estrogen receptor modulator (SERM), exhibits antimycobacterial activity and inhibits M. tuberculosis growth in macrophages. However, it remains unknown whether such antimicrobial activity is a general property of all SERMs and how it works. In this study, we identified that bazedoxifene (BZA), a newer SERM, inhibits intracellular M. tuberculosis growth in macrophages. BZA treatment increases autophagosome formation and LC3B-II protein expression in M. tuberculosis-infected macrophages. We further demonstrated that the enhancement of autophagy by BZA is dependent on increased reactive oxygen species (ROS) production and associated with phosphorylation of Akt/mTOR signaling. In summary, our data reveal a previously unappreciated antimicrobial function of BZA and suggest that future investigation focusing on the mechanism of action of SERMs in macrophages may lead to new host-directed therapies against TB.

**IMPORTANCE** Since current strategies for the treatment of multidrug-resistant tuberculosis (MDR-TB) and extensively drug-resistant tuberculosis (XDR-TB) have low efficacy and highly negative side effects, research on new treatments including novel drugs is essential for curing drug-resistant tuberculosis. Host-directed therapy (HDT) has become a promising idea to modulate host cell responses to enhance protective immunity against pathogens. Bazedoxifene (BZA), which belongs to a new generation of SERMs, shows the ability to inhibit the growth of M. tuberculosis in macrophages and is associated with autophagy. Our findings reveal a previously unrecognized antibacterial function of BZA. We propose that the mechanism of SERMs action in macrophages may provide a new potential measure for host-directed therapies against TB.

## Abstract (precis)

Since current strategies for the treatment of multidrug-resistant tuberculosis (MDR-TB) and extensively drug-resistant tuberculosis (XDR-TB) have low efficacy and highly negative side effects, research on new treatments including novel drugs is essential for curing drug-resistant tuberculosis. Host-directed therapy (HDT) has become a promising idea to modulate host cell responses to enhance protective immunity against pathogens. Bazedoxifene (BZA), which belongs to a new generation of SERMs, shows the ability to inhibit the growth of M. tuberculosis in macrophages and is associated with autophagy. Our findings reveal a previously unrecognized antibacterial function of BZA. We propose that the mechanism of SERMs action in macrophages may provide a new potential measure for host-directed therapies against TB.

## INTRODUCTION

Tuberculosis (TB), a chronic infectious disease caused by Mycobacterium tuberculosis, remains as a leading killer worldwide. In 2018, there were more than 10 million new cases and 1.57 million deaths from TB occurred (1). Although current antibiotics are effective at treating the majority of active TB cases, there is still a relapse rate of up to 9% even after completion of a 6- to 9-month treatment regimen (2, 3). Moreover, the emergence of drug-resistant TB (4), as in the case of multiple-drug-resistant TB (MDR-TB) and extensively drug-resistant TB (XDR-TB) (5), has further impeded the effectiveness of the current regimen. This situation has led to the emergence of a new paradigm in TB drug discovery that involves therapeutic modulation of host cell functions in order to improve pathogen eradication, namely, host-directed therapy (HDT) (6). Since the mechanisms underlying HDT are completely different from those for antibiotics, which directly kill the mycobacteria, HDT has become an attractive strategy to shorten treatment regimens and to treat TB patients infected with drug-resistant M. tuberculosis strains (6, 7).

Tamoxifen (TAM), known as a selective estrogen receptor modulator (SERM), is widely used for the treatment of breast cancer (8). It has been initially suggested to have antimycobacterial activity using the computationally predicted “TB drugome” approach—a method for drug repositioning for tuberculosis treatment (9). In line with this, a recent study revealed that TAM exhibits antimycobacterial activity against drug-resistant M. tuberculosis and inhibits intracellular M. tuberculosis growth in macrophages (10). However, the underlying mechanism remains largely unknown. In particular, it is uncertain whether inhibition of intracellular M. tuberculosis growth by TAM is due to direct killing of bacteria or to modulation of bactericidal activity of macrophages. In addition, it remains to be elucidated whether other SERMs exhibit similar antimycobacterial effects in human macrophages.

In this study, we investigated whether bazedoxifene (BZA), a newer SERM that is effective in TAM-resistant breast cancer and has a high safety profile (11), has an antimycobacterial effect. We found that BZA efficiently inhibits intracellular M. tuberculosis growth in human macrophages.

As well, the autophagic machinery is a fundamental cellular response to defense against infectious pathogens, including M. tuberculosis infections (12). During autophagy, M. tuberculosis is sequestered within a double-membrane cytosolic vesicle called the autophagosome (13). After that, autophagosomes mature into autolysosomes through fusion with lysosomes, which leads to the digestion and death of the microbes (14) and subsequently activates the innate and adaptive immunity (15–17). In this study, we also identified that BZA inhibition is associated with enhanced autophagy.

## RESULTS

### BZA inhibits intracellular M. tuberculosis growth in macrophages.

**FIG 1:**
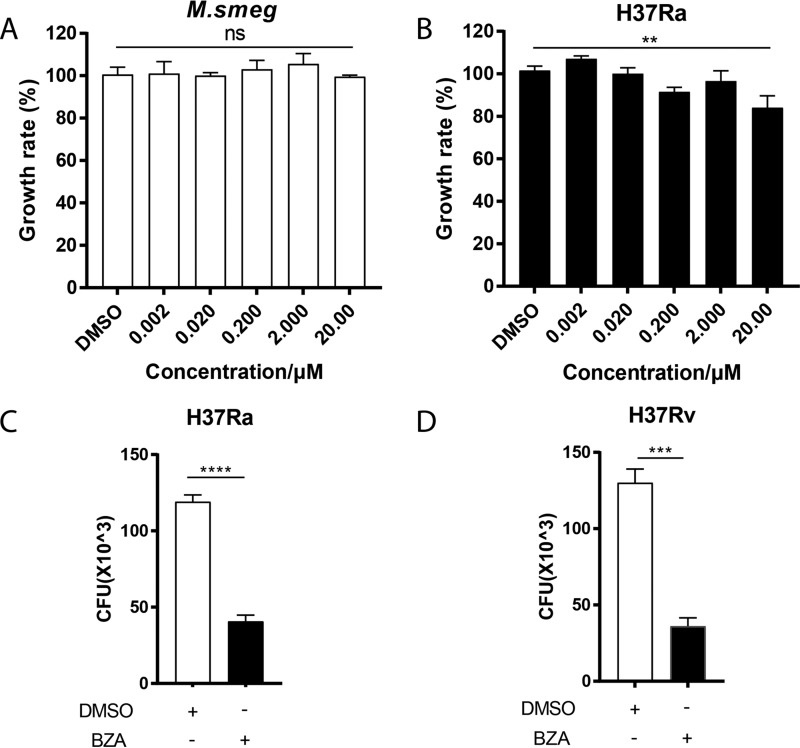
The inhibition effect of BZA on M. tuberculosis growth *in vitro* and *in vivo*. (A and B) Bacteria were diluted with liquid 7H9 culture medium to an OD of 0.01 and transferred to a 96-well microtiter plate. Bacteria were grown in the presence of 10-fold serially diluted concentrations of BZA. DMSO was added as a negative control. The growth rate of M. smegmatis (A) and M. tuberculosis H37Ra (B) was calculated. (C and D) CFU of H37Ra (C) and H37Rv (D) (MOI = 10:1) treated with BZA (5 μM) is shown. THP-1 macrophages were differentiated by PMA and infected with H37Ra (MOI = 10:1) for 6 h. After washing three times with prewarmed sterile phosphate-buffered saline (PBS) to remove extracellular bacteria, the infected THP-1 cells were treated with or without BZA (5 μM) for another 72 h. Cells were lysed in 0.1% SDS and plated on 7H10 plates. The bactericidal activity of macrophages was assessed by determining CFU of intracellular H37Ra and H37Rv. The data represent means ± standard deviations (SD) for three independent experiments. Values that are significantly different by one-way ANOVA and two-tailed Student’s *t* test are indicated by asterisks as follows: *, *P* < 0.05; **, *P* < 0.01; ***, *P* < 0.001; ****, *P* < 0.0001 (ns, not significant).

To investigate whether BZA has a direct antimycobacterial activity similar to TAM, we treated Mycobacterium smegmatis and M. tuberculosis H37Ra with BZA at different concentrations directly. Our data showed that BZA did not inhibit the growth of M. smegmatis and M. tuberculosis H37Ra at concentrations up to 20 μM (10.613 μg/ml) (Fig. 1A). Although the growth rate of M. tuberculosis H37Ra was statistically different from the control group when the concentration was up to 20 μM, the bacterial growth inhibition efficiency was very low, less than 20% (Fig. 1B). While exhibiting a moderate inhibitory effect on M. smegmatis and M. tuberculosis H37Ra growth at a concentration higher than 200 μM, BZA at this concentration caused significant cytotoxicity with 52% cell viability (see Fig. S1 in the supplemental material).

10.1128/mSphere.00124-20.1 FIG S1 The direct antituberculosis effect and cytotoxic effect on macrophages regulated by BZA. (A and B) Bacteria were diluted with liquid 7H9 culture medium to an OD of 0.01 and transferred to a 96-well microtiter plate. BZA was incubated with bacteria at a 200 μM concentration. DMSO was added as negative control. The growth rates of M. smegmatis and M. tuberculosis H37Ra were calculated. (C) PMA-differentiated THP-1 macrophages were treated with different concentration of BZA (200 μM, 20 μM, and 2 μM), and DMSO was added as negative control. Cells were cultured at 37°C for 72 h. The WST-1 reagent kit (Beyotime, Shanghai, China) was used to measure cell viability. Values that are significantly different by one-way ANOVA and two-tailed Student’s *t* test are indicated by asterisks as follows: *, *P* < 0.05; **, *P* < 0.01; ***, *P* < 0.001; ****, *P* < 0.0001. Download FIG S1, TIF file, 2.2 MB. Copyright © 2020 Ouyang et al. 2020 Ouyang et al. This content is distributed under the terms of the Creative Commons Attribution 4.0 International license.

We next investigated whether BZA could inhibit M. tuberculosis growth indirectly through enhancing macrophage bactericidal activity. We observed that when used at a dose (5 μM) that did not display apparent inhibitory effect on M. tuberculosis growth in 7H9 medium, BZA significantly inhibited both M. tuberculosis H37Ra and H37Rv growth in THP-1 macrophages as determined by counting CFU at 72 h postinfection (Fig. 1C and D). These results indicated that BZA significantly suppresses intracellular M. tuberculosis growth through modulating macrophage functions.

### BZA inhibits intracellular M. tuberculosis growth in macrophages through enhancing autophagy.

**FIG 2:**
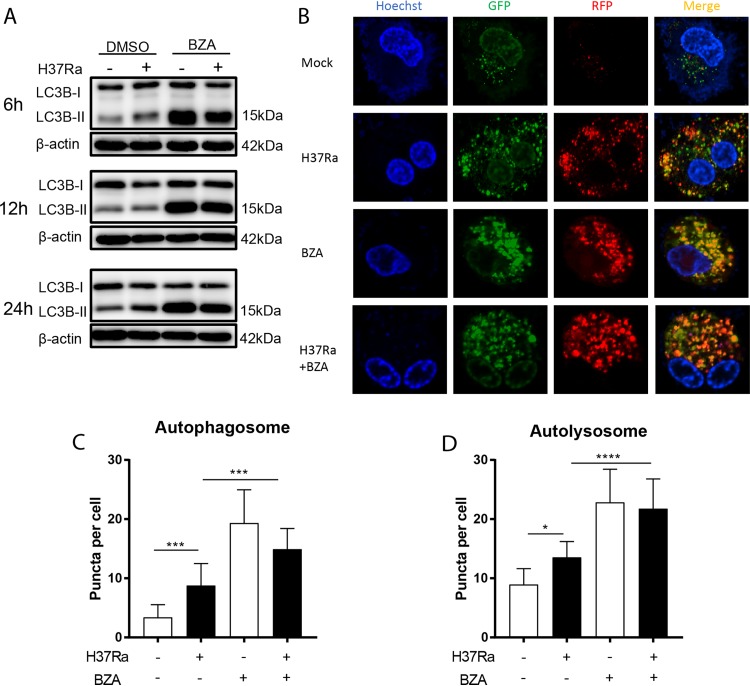
BZA induced LC3B-II protein and increased the formation of autophagosomes and autolysosomes in macrophages. (A) PMA-differentiated THP-1 macrophages were infected with H37Ra (MOI = 10:1) for 6, 12, and 24 h in the presence or absence of BZA (5 μM). The LC3B-II protein level was analyzed by Western blotting. (B) mRFP-GFP-LC3B reporter THP-1 cells were differentiated by PMA and infected with H37Ra (MOI = 10:1) with or without BZA (5 μM) for 12 h. Representative confocal microscopy images are shown (bars, 5 μm). (C and D) The autophagosome puncta (yellow) per cell (C) and the autolysosome puncta (red) per cell (D) were calculated. The data represent the means ± standard deviations (SD) for three independent experiments. Values that are significantly different by one-way ANOVA are indicated by asterisks as follows: *, *P* < 0.05; **, *P* < 0.01; ***, *P* < 0.001; ****, *P* < 0.0001.

We next investigated the mechanism underlying BZA inhibition of intracellular M. tuberculosis growth. Since autophagy is critical for macrophages to inhibit M. tuberculosis growth (18, 19) and previous studies have demonstrated that BZA induces autophagy in cancer cells (20), we first investigated the effect of BZA on autophagy induction. As expected, we found that BZA treatment significantly increased autophagy in macrophages, regardless of M. tuberculosis infection, as evident from a significantly increased conversion of soluble LC3B-I to lipid bound LC3B-II (Fig. 2A and Fig. S2A). To further examine the effect of BZA on autophagy flux, we monitored LC3B aggregation in autophagosomes and autolysosomes using the stably transformed monomeric red fluorescent protein (mRFP)-green fluorescent protein (GFP)-LC3 reporter THP-1 cells. Our result shows that BZA treatment significantly increased autophagosome and autolysosome forming punctate in H37Ra-infected cells compared with their mock-treated counterparts. (Fig. 2B to D), suggesting that enhanced autophagy contributes to BZA-mediated suppression of intracellular M. tuberculosis growth.

10.1128/mSphere.00124-20.2 FIG S2 Suppression of autophagosome and autophagolysosomal fusion increases BZA-induced macrophage LC3B-II accumulation. (A) PMA-differentiated THP-1 macrophages were infected with H37Rv (MOI = 10:1) for 24 h in the presence or absence of BZA (5 μM). The LC3B-II protein level was analyzed by Western blotting. (B and C) PMA-differentiated THP-1 macrophages were infected with H37Ra (MOI = 10:1) (B) and H37Rv (MOI = 10:1) (C) for 24 h with or without BZA (5 μM) and Baf (100 nM). The LC3B-II protein level was analyzed by Western blotting. Download FIG S2, TIF file, 2.8 MB. Copyright © 2020 Ouyang et al. 2020 Ouyang et al. This content is distributed under the terms of the Creative Commons Attribution 4.0 International license.

**FIG 3:**
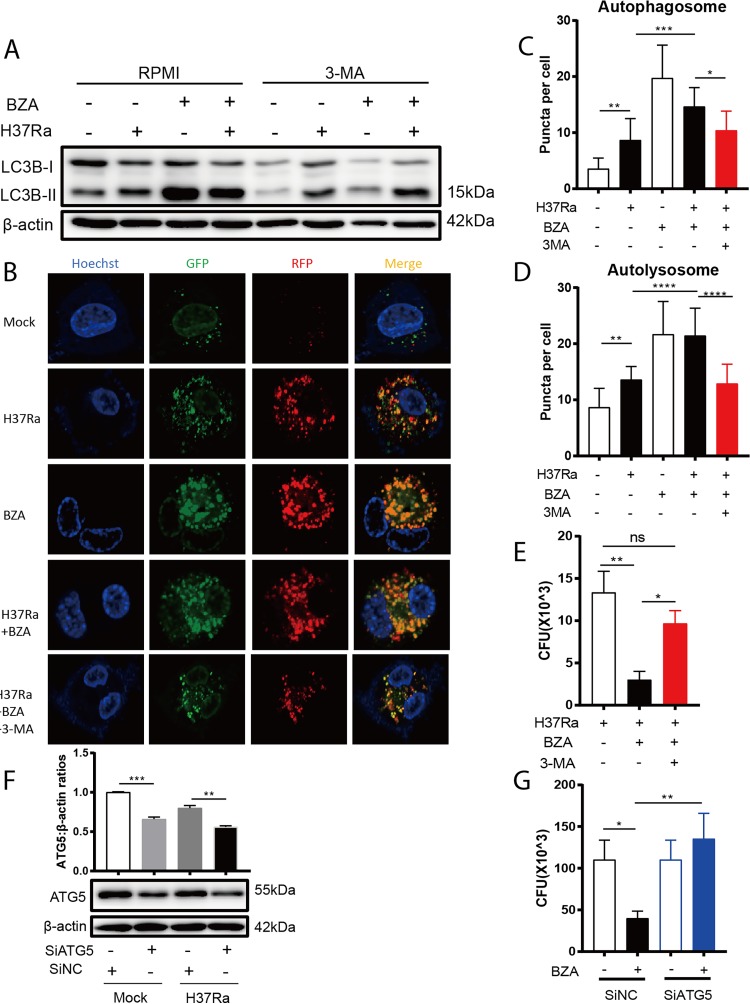
Inhibiting autophagy-mediated impairment of BZA induced bacterial activity against M. tuberculosis in macrophages. (A) PMA-differentiated THP-1 macrophages were infected with H37Ra (MOI = 10:1) for 24 h in the presence or absence of BZA (5 μM) and 3-MA (10 mM). The LC3B-II protein level was analyzed by Western blotting. (B) mRFP-GFP-LC3B reporter THP-1 cells were differentiated by PMA and infected with H37Ra (MOI = 10:1) with or without BZA (5 μM) and 3-MA (10 mM) for 12 h. Representative confocal microscopy images are shown (bars, 5 μm). (C and D) The autophagosome puncta (yellow) per cell (C) and the autolysosome puncta (red) per cell (D) were calculated. (E) PMA-differentiated THP-1 macrophages were infected with H37Ra (MOI = 10:1) for 6 h. After washing three times with prewarmed sterile phosphate-buffered saline (PBS) to remove extracellular bacteria, the infected THP-1 cells were treated with or without BZA (5 μM) and 3-MA (10 mM) for another 72 h. Cells were lysed in 0.1% SDS and plated on 7H10 plates. The bactericidal activity of macrophages was assessed by determining CFU of intracellular H37Ra. (F) PMA-differentiated THP-1 macrophages were transfected with *ATG5* small interfering RNA (siRNA) for 36 h. Scrambled siRNA was used as a negative control. These macrophages were then infected with H37Ra (MOI = 10:1) and treated with BZA (5 μM) as described above. The ATG5 protein level was analyzed by Western blotting. ATG5/β-actin ratios are shown above the protein band. (G) The bactericidal activity of macrophages treated as described above was assessed by CFU. The data represent the means ± standard deviations (SD) for three independent experiments. Values that are significantly different by one-way ANOVA are indicated by asterisks as follows: *, *P* < 0.05; **, *P* < 0.01; ***, *P* < 0.001; ****, *P* < 0.0001 (ns, not significant).

To determine the causal relationship between the effect of BZA on intracellular M. tuberculosis growth and its role in inducing autophagy, bafilomycin A1 (Baf), an autophagy inhibitor that prevents maturation of autophagic vacuoles by inhibiting fusion between autophagosomes and lysosomes (21), was used to block autophagy induced by BZA. As expected, the cumulative amount of LC3B-II increased after using Baf in H37Ra- or H37Rv-infected macrophages treated with BZA (Fig. S2B and C), indicating that the increased accumulation of LC3B-II promoted by BZA is not due to the inhibition of downstream autophagic flow. Another autophagy inhibitor, 3-methyladenine (3-MA), a phosphatidylinositol 3-kinase (PI3K) inhibitor (22), was also used to block autophagy in the presence of BZA, and intracellular M. tuberculosis load was determined by CFU counting. As expected, 3-MA significantly reduced BZA-induced autophagy as determined by immunoblotting of LC3B-II conversion (Fig. 3A) as well as by counting and analyzing autophagosome and autolysosome formation in mRFP-GFP-LC3B reporter cells (Fig. 3B to D). Importantly, the inclusion of 3-MA almost completely abrogated BZA-mediated suppression of M. tuberculosis growth within macrophages (Fig. 3E), indicating the effect of BZA on intracellular M. tuberculosis growth was dependent on its role in regulating autophagy.

To confirm the role of autophagy in BZA-mediated suppression of intracellular M. tuberculosis growth, we knocked down ATG5, a key protein in autophagy initiation and processing (23), and analyzed the effect of BZA on macrophage bactericidal activity. As demonstrated in Fig. 3F, ATG5 expression was significantly reduced following the treatment with ATG5-specific small interfering RNA (siRNA) for 36 h (Fig. 3F). In agreement with 3-MA treatment experiments, we found that ATG5 knockdown almost completely abolished BZA-mediated inhibition of intracellular growth of M. tuberculosis (Fig. 3G). However, we did not observe a significant increase of CFU in the siATG5 group compared to the control (Fig. 3G), which might be due to the knockdown efficiency, as a previous study showed that the complete knockout of ATG5 showed only a slight increase in CFU compared to wild-type (WT) macrophages (24). Taken together, these data confirmed that BZA suppresses M. tuberculosis growth in macrophages through enhancing autophagosome formation.

### BZA-induced autophagy is associated with ROS production and phosphorylation of Akt/mTOR signal.

**FIG 4:**
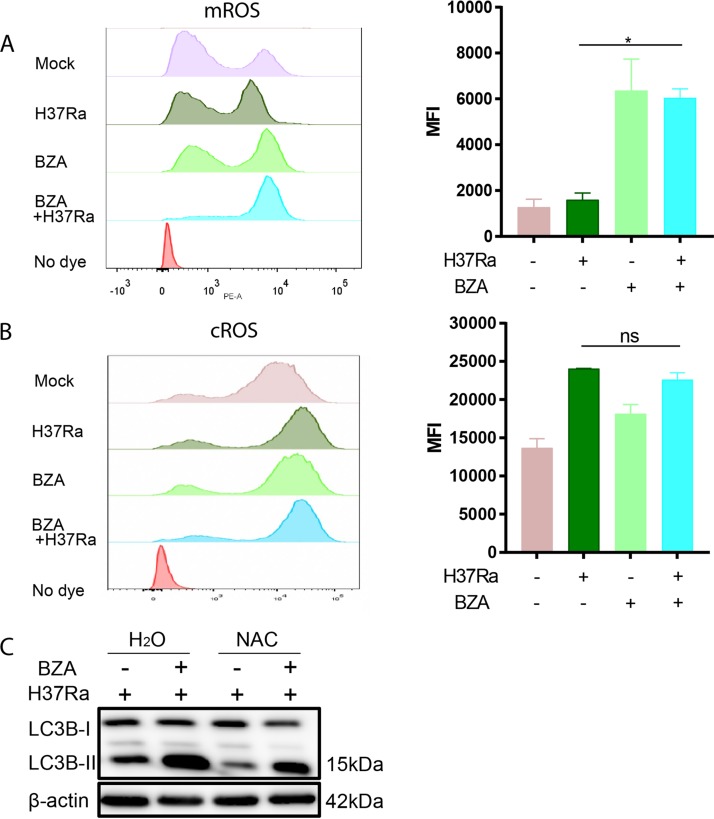
BZA increased M. tuberculosis-induced reactive oxygen species generation, and NAC reduced autophagy level in macrophages. (A and B) The mROS (A) and cROS (B) levels in H37Ra-infected THP-1 macrophages with 48-h BZA (5 μM) treatment were detected using MitoSOX (10 μM) probes and H2DCF-DA (10 μM) probes. Mean fluorescence intensity (MFI) of ROS was analyzed by flow cytometry. (C) THP-1 macrophages were differentiated by PMA and infected with H37Ra (MOI = 10:1) for 24 h in the presence or absence of BZA (5 μM) and NAC (5 mM). The LC3B-II protein level from H37Ra-infected THP-1 macrophages with or without BZA (5 μM) and NAC (5 mM) treatment was analyzed by Western blotting. The data represent the means ± standard deviations (SD) for three independent experiments. Values that are significantly different by one-way ANOVA are indicated by asterisks as follows: *, *P* < 0.05; **, *P* < 0.01 (ns, not significant).

To determine the mechanisms by which the BZA modulates macrophage bactericidal capability against M. tuberculosis, we also investigated the effect of BZA on M. tuberculosis-induced reactive oxygen species (ROS) production, one of the pivotal mechanisms for the killing of intracellular M. tuberculosis by macrophages (25). We found that mitochondrial ROS (mROS) production (Fig. 4A) was significantly increased in M. tuberculosis-infected macrophages upon treatment with BZA at 24 h postinfection, whereas cytoplasmic ROS (cROS) was not significantly changed (Fig. 4B).

It has been reported that mitochondrial ROS is an important mediator of autophagy (26, 27). We therefore used *N*-acetyl-l-cysteine (NAC), a ROS-scavenging agent, in the presence of BZA treatment to test whether increased mROS contributes to enhanced autophagy induced by BZA in M. tuberculosis-infected macrophages. In line with previous reports (28, 29), we found that addition of NAC abolished the ability of BZA to induce autophagy (Fig. 4C).

**FIG 5:**
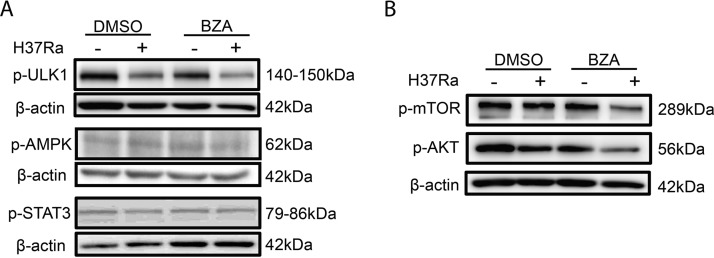
BZA reduced Akt/mTOR levels in macrophages. THP-1 macrophages were differentiated by PMA and infected with H37Ra (MOI = 10:1) for 24 h in the presence or absence of BZA (5 μM) and NAC (5 mM). The phospho-ULK, phospho-AMPK, and phospho-STAT3 protein levels (A) and phospho-mTOR and phospho-Akt protein levels (B) from H37Ra-infected THP-1 macrophages with or without BZA (5 μM) and NAC (5 mM) treatment were analyzed by Western blotting.

Phosphorylation of mTOR and Akt is a key signaling step for the initiation of autophagy (30, 31). To gain an insight into the signaling for BZA-induced autophagy, we determined the phosphorylation of mTOR, Akt, ULK, AMPK, and STAT3. There was no difference in the extent of ULK, AMPK, and STAT3 phosphorylation between the presence and absence of BZA treatment (Fig. 5A). In contrast, the levels of phospho-mTOR and phospho-Akt in BZA-treated M. tuberculosis-infected macrophages were significantly reduced (Fig. 5B) in comparison with those without BZA treatment. These results imply that BZA-induced autophagy is associated with phosphorylation of mTOR and Akt signaling.

## DISCUSSION

With the emergence of drug-resistant TB, there is an urgent need for new TB treatment strategies. Repurposed FDA-approved drugs for anti-TB therapy are an attractive approach due to the safety profile of the approved products. In this study, we show that BZA, a newer SERM, significantly inhibits M. tuberculosis growth in human macrophages. This effect of BZA is mediated through enhancing autophagy in infected macrophages. We propose that BZA treatment increases mROS production and promotes autophagosome formation in macrophages, which ultimately enhances the passage of M. tuberculosis into the phagolysosome for pathogen destruction.

BZA is an indole-based estrogen receptor (ER) ligand with a unique structure, which differs from TAM and raloxifene (32) but exhibits similar functions (33). BZA is well tolerated and possesses improved inhibitory potency against the Y537S and D538G ERa mutants compared to TAM (34). In contrast to previous reports that TAM has direct antimycobacterial activity at a low concentration (16 μM) (10), we found that BZA displays only moderate antimycobacterial activity in 7H9 culture medium at a high concentration (200 μM). Therefore, these findings suggest that TAM and BZA limit mycobacterial growth through both shared and distinct mechanisms. The exact reason underlying the difference between TAM and BZA in extracellular bacterial killing is currently unknown, although this could be due simply to the intrinsic differences in their chemical structure. However, consistent with TAM and raloxifene (10, 35, 36), we found that BZA significantly enhanced the macrophage bactericidal activity against M. tuberculosis. More importantly, we confirmed that this effect of BZA is achieved through regulating autophagy activity in macrophages. By simultaneously analyzing the amount of LC3B-II and the formation of the autophagosome and autolysosome, we inferred that BZA facilitates autophagic activity and therefore enhances the elimination of intracellular M. tuberculosis via autophagosome synthesis and lysosome degradation.

How autophagy is regulated in M. tuberculosis-infected macrophages is complex. It has been reported that ROS can induce autophagy (37), one means of which is through the inhibition of Akt signaling, and mTOR (38). In line with this report, we found that mROS production was elevated after BZA treatment in M. tuberculosis-infected macrophages and that depletion of mROS by NAC abolished BZA-induced autophagy. However, our data have not addressed the exact signal pathway from ROS to autophagy during BZA treatment. Although we demonstrated that the disruption of autophagy abolished the effect of BZA and that BZA-induced autophagy was dependent on the upregulation of mROS production, we cannot exclude and discriminate the contribution of direct killing bacteria by mROS.

In summary, we have demonstrated that BZA significantly inhibits intracellular M. tuberculosis growth in macrophages through enhancing ROS production and autophagy. These findings suggest that BZA may be considered a host-directed drug candidate for TB treatment and that further *in vivo* investigations are warranted.

## MATERIALS AND METHODS

### Bacterial strains and culture conditions.

M. smegmatis mc^2^155 and M. tuberculosis strains H37Ra and H37Rv were used. The bacteria were grown in Middlebrook 7H9 broth (BBL Microbiology Systems) supplemented with 10% oleic acid-albumin-dextrose-catalase (OADC; Becton, Dickinson), 0.05% Tween 80 (Sigma), and 0.2% glycerol (Sigma), for 5 to 7 days at 37°C with shaking to achieve mid-logarithmic phase (optical density at 600 nm [OD_600_] = 0.3 to 0.8).The bacteria were resuspended in serum-free RPMI medium and sonicated to obtain a single-cell suspension before being used in experiments. The concentration of bacteria was determined by the OD_600_ as a function of CFU per milliliter.

### Cell culture and chemicals.

Cells of the human monocytic cell line THP-1 were purchased from the Cell Bank of the Chinese Academy of Sciences (Shanghai, China). THP-1 cells and THP-1 cells transformed with an mRFP-GFP-LC3B reporter (provided by Hongbo Shen, Institute Pasteur of Shanghai, China) were maintained in RPMI 1640 (Corning) culture medium supplemented with 10% fetal bovine serum (FBS; Invitrogen, Life Technologies) at 37°C and 5% CO_2_. The THP-1 cells were seeded at 5 × 10^5^ cells/ml in a 12-well plate in complete RPMI 1640 and differentiated using phorbol 12-myristate 13-acetate (PMA; Sigma, P8139) at 40 ng/ml for 24 h. Following removal of the PMA-containing medium, the cells were incubated in the fresh complete RPMI 1640 medium for 12 h at 37°C for further use.

Bazedoxifene (acetate) (BZA) was purchased from MedChemExpress (MCE; China), and a 10 mM stock solution was dissolved and diluted in dimethyl sulfoxide (DMSO; Sigma, USA). Bafilomycin A1 (Baf), *N*-acetyl-l-cysteine (NAC), and 3-methyladenine (3-MA) were purchased from Sigma-Aldrich (Sigma, USA). The final concentration of DMSO in treatment did not exceed 0.1% (vol/vol).

### Extracellular H37Ra survival determination.

*In vitro* antimycobacterial activity was tested with M. smegmatis and M. tuberculosis H37Ra. The bacteria were diluted with 7H9-OADC to an OD of 0.01 and transferred to a 96-well microtiter plate for the survival assay. Then, BZA with a serial dilution of 10-fold in 7H9 medium was added to the bacterial culture. DMSO was added as a negative control. The bacterial plates were incubated at 37°C with horizontal shaking (110 rpm). The absorbance (OD_600_) was measured with an Epoch 2 microplate spectrophotometer (BioTek, USA) at indicated times, and the growth rate of bacteria was calculated.

### Cell viability and proliferation assay.

THP-1 cells (5 × 10^3^ cells/well) were incubated in a 96-well plate with 40 ng/ml PMA for 24 h. Cells were then maintained in fresh prewarmed complete RPMI 1640 for 12 h at 37°C for further use. Different concentration of BZA (200 μM, 20 μM, and 2 μM) and DMSO, as a negative control, were added and cultured for an additional 72 h. WST-1 reagent (Beyotime, Shanghai, China) was added into the well (10 μl/well) and incubated at 37°C for 2 h to measure cell viability and proliferation. The absorbance was measured at 450 nm and 690 nm with an Epoch 2 microplate spectrophotometer (BioTek, USA), and cell viability was calculated according to the manufacturer’s instructions.

### CFU assays.

PMA-differentiated THP-1 macrophages (5 × 10^5^ cells) were infected with two laboratory M. tuberculosis strains (H37Ra and H37Rv) at a multiplicity of infection (MOI) of 10 for 6 h at 37°C, 5% CO_2_. After washing three times with prewarmed sterile phosphate-buffered saline (PBS) to remove extracellular bacteria, the infected THP-1 cells are treated with or without BZA (5 μM) for another 72 h. Cells were washed three times with PBS and then lysed with 500 μl of PBS containing 0.1% SDS. Triple experimental groups for each treatment were plated on Middlebrook 7H10 agar (Difco; Middlebrook) supplemented with 10% OADC and incubated vertically at 37°C for 3 weeks, and then the colonies were counted. CFU obtained from two or three dilutions was used to calculate the total number of CFU per milliliter. Data were presented as the amount of M. tuberculosis in 5 × 10^5^ cells and the percentage of mycobacterial survival in BZA-treated and untreated cells.

### Measurement of H37Ra in lysosomes and colocalization with LC3B.

mRFP-GFP-LC3B reporter THP-1 macrophages (1 × 10^5^ cells/ml) were differentiated using PMA and infected with mycobacterial strain H37Ra (MOI of 10:1) for 6 h. After washing with PBS, the cells were cultured with or without BZA (5 μM) for 12 h. The cells were then washed twice in PBS and with 4% paraformaldehyde solution (Solarbio, China) for 15 min at room temperature. Cells were stained with Hoechst stain and mounted with antifade agent (Invitrogen, USA). The cells were visualized, and images were acquired with an Olympus FV1000 confocal microscope (Nikon A1R) and processed using Image J software (NIH, USA). A total of 30 to 40 infected cells were analyzed, and the level of autophagy was measured by enumerating the number of LC3 puncta per cell.

### Measurement of ROS.

PMA-differentiated THP-1 macrophages (5 × 10^5^ cells/ml) were cultured in 12-well plates and infected with H37Ra (MOI of 10:1) for 6 h. Extracellular bacteria were removed by washing 3 times with prewarmed sterile PBS. The cells were cultured in fresh medium with or without BZA (5 μM) for 24 h. The generation of cytoplasmic ROS (cROS) was quantified using 5- (and -6)-chloromethyl-2′,7′-dichlorodihydrofluorescein diacetate, acetyl ester (CM-H2DCF-DA) (Invitrogen, USA), according to the manufacturer’s instructions. The generation of mitochondrial ROS (mROS) was quantified using MitoSOX red mitochondrial superoxide indicator (Invitrogen, USA). The cells were incubated with H2DCF-DA (10 μM) and MitoSOX (10 μM) for 15 min at 37°C in the dark. All fluorescence intensities of cells were measured by flow cytometry (BD FACSAria II flow cytometer) and analyzed by FlowJo 6.1 according to the manufacturer’s protocol.

### RNA interference and transfection.

PMA-differentiated THP-1 macrophages (5 × 10^5^ cells/ml) were transfected with *ATG5* siRNA (RiboBio; siB12531154855-1-5; China) using Lipofectamine RNAiMAX (ThermoFisher, USA) according to the manufacturer’s protocol. The siRNA and Lipofectamine complexes were prepared in Opti-MEM (Gibco) at a 1:1 ratio. Negative control (NC) vector without any insert was used as a negative control. The medium was replaced after 36 h, cells were washed twice with PBS before lysing, and the efficiency of knockdown was determined by Western blotting. For the CFU test, THP-1 cells were infected with H37Ra (MOI of 10:1) for 6 h after 36 h of treatment with siRNA silencing. Extracellular bacteria were washed 3 times with prewarmed serum-free RPMI and treated with BZA for 72 h.

### Western blot analysis.

PMA-differentiated THP-1 macrophages (5 × 10^5^ cells/ml) were cultured in 12-well plates and infected with H37Ra (MOI of 10:1) with or without BZA (5 μM) for 6, 12, or 24 h. After washing with PBS, the cells were lysed in RIPA lysis buffer (Cell Signaling Technology). The protein concentration of the resultant lysates was measured with a bicinchoninic acid (BCA) protein kit (Beyotime, China). The protein samples were boiled for 10 min. Equal amounts of proteins were adjusted according to the molecular weight of the protein, electrophoresed on SDS-PAGE gels, and then transferred onto a polyvinylidene difluoride membrane (Merck/Millipore). The membrane was blocked with 5% skim milk powder (BD) solution in PBS with Tween 20 (PBST) for 1 to 2 h at room temperature and then incubated with antibodies against LC3B (Sigma, USA), p-mTOR (Abcam, United Kingdom), p-Akt (Abcam, United Kingdom), actin (Abcam, United Kingdom), p-ULK (Cell Signaling Technology, USA), p-AMPKa (Cell Signaling Technology, USA), p-STAT3 (Cell Signaling Technology, USA), and ATG5 (Cell Signaling Technology, USA) at 4°C for 12 h. After washing with 1× PBST for 10 min, the membrane was incubated with appropriate secondary antibody, goat anti-rabbit IgG–horseradish peroxidase (HRP) (Abcam, USA). After washing with 1× PBST, the protein band was visualized by enhanced chemiluminescence (ECL) detection solution (ThermoFisher). The digital images of the protein bands were acquired using an ImageQuant LAS 4000 (GE Healthcare Bio-Science) system.

### Statistical analysis.

Statistical analysis was performed using GraphPad Prism 7 software (GraphPad Software, Inc.). Two-tailed Student’s *t* test was used to evaluate the measurement of one parameter. One-way analysis of variance (ANOVA) with multiple comparisons was used to analyze more than two groups. Data were expressed as mean ± standard deviation (SD). Differences between groups were considered statistically significant when *P* was <0.05.
