# Supplementary figures and images for: Bazedoxifene Suppresses Intracellular Mycobacterium tuberculosis Growth by Enhancing Autophagy
Source: mSphere. 2020 Apr 8;5(2):e00124-20. doi: 10.1128/mSphere.00124-20 (PMC7142296; doi:10.1128/mSphere.00124-20)

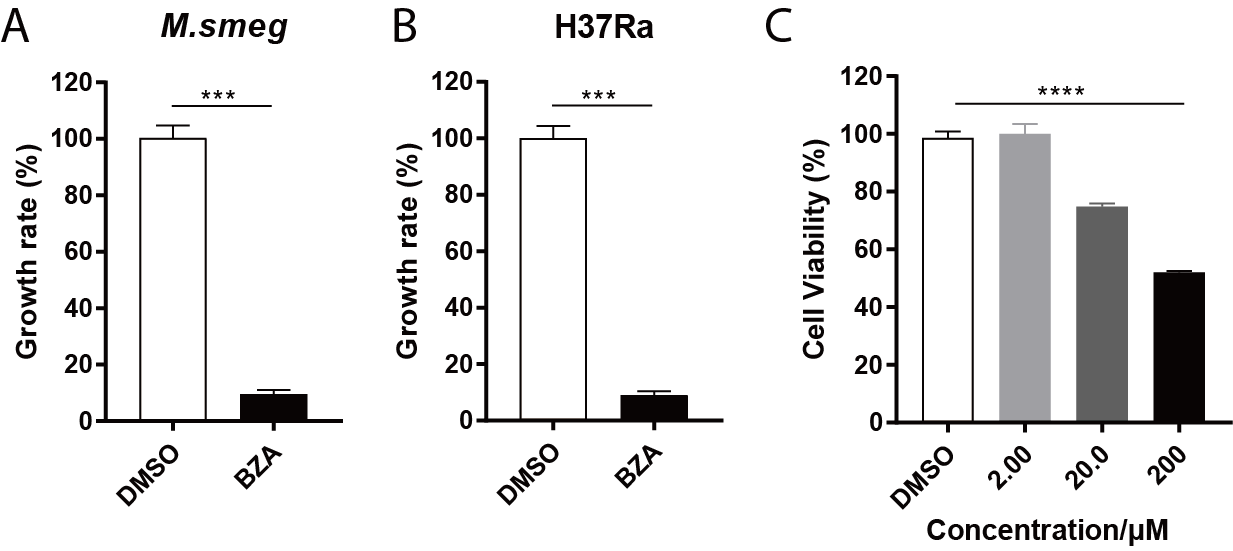

Supplement: FIG S1 [file mSphere.00124-20-sf001.tif]

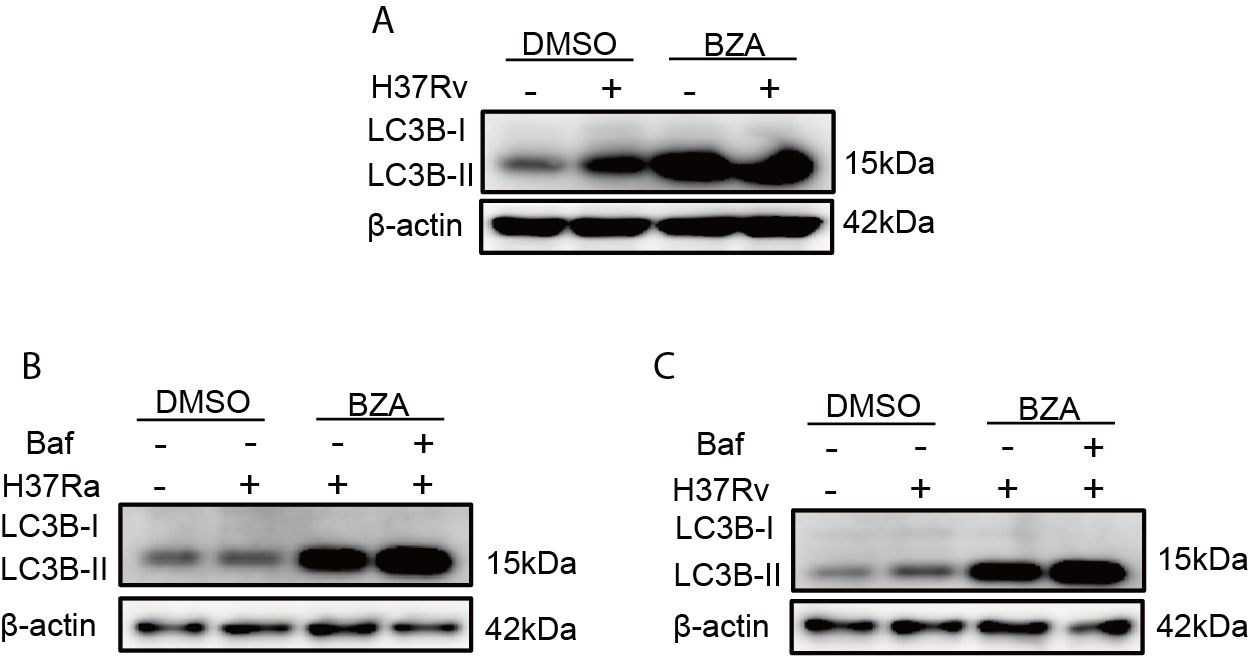

Supplement: FIG S2 [file mSphere.00124-20-sf002.tif]
